# Diffusion-Weighted Imaging in Oncology: An Update

**DOI:** 10.3390/cancers12061493

**Published:** 2020-06-08

**Authors:** Carmelo Messina, Rodolfo Bignone, Alberto Bruno, Antonio Bruno, Federico Bruno, Marco Calandri, Damiano Caruso, Pietro Coppolino, Riccardo De Robertis, Francesco Gentili, Irene Grazzini, Raffaele Natella, Paola Scalise, Antonio Barile, Roberto Grassi, Domenico Albano, on behalf of the Young SIRM Working Group

**Affiliations:** 1 IRCCS Istituto Ortopedico Galeazzi, 20161 Milano, Italy; carmelomessina.md@gmail.com; 2 Dipartimento di Scienze Biomediche per la Salute, Università degli Studi di Milano, 20133 Milano, Italy; 3 Radiology Unit, University of Palermo, 90127 Palermo, Italy; rodolfobig86@gmail.com (R.B.); bruno-alberto@hotmail.it (A.B.); 4 Department of Experimental, Diagnostic and Specialty Medicine-DIMES, University of Bologna, S.Orsola-Malpighi Hospital, 40126 Bologna, Italy; antobruno@hotmail.it; 5 Department of Biotechnology and Applied Clinical Sciences, University of L’Aquila, 67100 L’Aquila, Italy; federico.bruno.1988@gmail.com (F.B.); antonio.barile@univaq.it (A.B.); 6 Radiology Unit, A.O.U. San Luigi Gonzaga di Orbassano, Department of Oncology, University of Torino, 10043 Turin, Italy; marco.calandri@unito.it; 7 Department of Radiological, Oncological and Pathological Sciences, “Sapienza” University of Rome, Sant’Andrea University Hospital, 00161 Rome, Italy; damiano.caruso@uniroma1.it; 8 Department of Medical Surgical Sciences and Advanced Technologies “G.F. Ingrassia”-Radiology I Unit, University Hospital “Policlinico-Vittorio Emanuele”, 95123 Catania, Italy; pietrocoppolino@hotmail.it; 9 Department of Radiology, Ospedale Civile Maggiore, Azienda Ospedaliera Universitaria Integrata Verona, 37134 Verona, Italy; riccardo.derobertis@hotmail.it; 10 Section of Radiology, Unit of Surgical Sciences, Department of Medicine and Surgery, University of Parma, 43126 Parma, Italy; francescogentili@gmail.com; 11 Department of Radiology, Section of Neuroradiology, San Donato Hospital, 52100 Arezzo, Italy; irene.grazzini@gmail.com; 12 Department of Precision Medicine, University of Campania “L. Vanvitelli”, 80138 Naples, Italy; raffaele.natella@gmail.com (R.N.); roberto.grassi2013@gmail.com (R.G.); 13 Department of Diagnostic Imaging, Pisa University Hospital, 56124 Pisa, Italy; scalisepl@gmail.com; 14 Department of Biomedicine, Neurosciences and Advanced Diagnostics, Section of Radiological Sciences, University of Palermo, 90127 Palermo, Italy

**Keywords:** magnetic resonance imaging, diffusion weighted imaging, apparent diffusion coefficient, oncologic imaging, cancer imaging

## Abstract

To date, diffusion weighted imaging (DWI) is included in routine magnetic resonance imaging (MRI) protocols for several cancers. The real additive role of DWI lies in the “functional” information obtained by probing the free diffusivity of water molecules into intra and inter-cellular spaces that in tumors mainly depend on cellularity. Although DWI has not gained much space in some oncologic scenarios, this non-invasive tool is routinely used in clinical practice and still remains a hot research topic: it has been tested in almost all cancers to differentiate malignant from benign lesions, to distinguish different malignant histotypes or tumor grades, to predict and/or assess treatment responses, and to identify residual or recurrent tumors in follow-up examinations. In this review, we provide an up-to-date overview on the application of DWI in oncology.

## 1. Introduction

The widespread use of diffusion weighted imaging (DWI) in oncologic imaging has led to a large number of studies and promising data towards the introduction of this sequence in routine magnetic resonance imaging (MRI) protocols for several tumors. DWI provides “functional” information regarding the free diffusivity of water molecules into intra/inter-cellular spaces that in tumors mainly depend on cellularity. Basically, it is an adapted T2-weigthed sequence with two symmetric diffusion-sensitizing gradients around the 180° refocusing pulse. Diffusion sensitivity is related to a specific parameter, namely, the “*b*-value,” which varies according to the gradient amplitude, duration of the gradient, and time between the two gradients. The restriction of water diffusion can be then quantitatively analyzed with the calculation of the apparent diffusion coefficient (ADC), which describes tissue signal attenuation with increasing *b*-values.

Today, DWI is routinely used in several clinical scenarios, besides still being a hot research topic: it has been tested in almost all cancers to differentiate malignant from benign lesions, to distinguish different malignant histotypes or tumor grades, to predict and/or assess treatment responses, and to identify residual or recurrent tumors in follow-up examinations. This paper provides an up-to-date overview on the application of DWI in oncology. In this narrative review, we reviewed the most recent and pivotal original articles and systematic reviews that investigated the role of DWI in cancer to highlight strengths and weaknesses of this sequence.

## 2. Neuro

**Figure 1:**
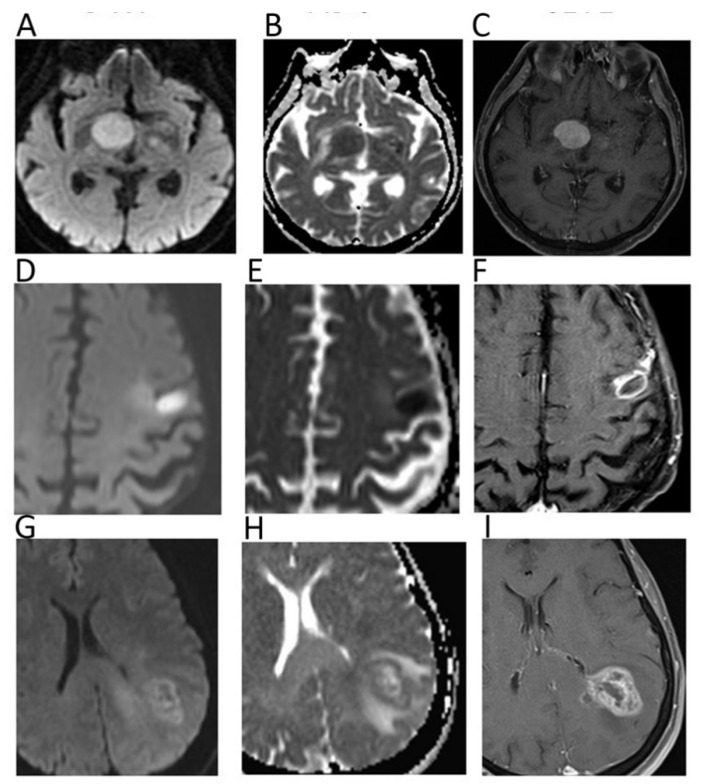
Diffusion weighted imaging (DWI) *b* = 1000 images (**A**,**D**,**G**), corresponding apparent diffusion coefficient (ADC) maps (**B**,**E**,**H**), and contrast–enhanced T1 w images (**C**,**F**,**I**) of patients with histologically proven primary central nervous system lymphoma (**A**–**C**), cerebral abscess (**D**–**F**), and glioblastoma (**G**–**I**). Mean ADC values in lymphoma (0.648 × 10^−3^ mm^2^/s) and in the central portion of the abscess (0.510 × 10^−3^ mm^2^/s) were lower than in glioblastoma. In particular, mean ADC values were higher in the necrotic core of the GBM (1.082 × 10^−3^ mm^2^/s) in comparison to the abscess.

DWI is included in routine brain tumor MRI protocol, generally including two *b*-values (*b* = 0, *b* = 1000 s/mm^2^). While conventional sequences are useful for mass detection, DWI helps achieving the diagnosis, providing information regarding tumor grade and type, and monitoring the treatment response [1]. DWI can differentiate abscesses, presenting impeded diffusion and low ADC (0.28–0.73 × 10^−3^ mm^2^/s), from necrotic tumors, showing higher ADC (Figure 1) [2].

With regard to tumor type, low ADC suggests a hypercellular malignancy such as lymphoma, which presents lower ADC compared to high-grade gliomas (HGGs) and metastases (Figure 1) [1]. The ADC of dysembryoplastic neuroepithelial tumors was reported to be higher than that of astrocytic tumors, while ADC of medulloblastoma is generally lower than that of ependymomas and posterior fossa astrocytomas [3]. Thus, ADC can be helpful in narrowing the differential diagnosis for both adult and paediatric intracranial masses. Due to their cellular content, low-grade gliomas usually present higher ADC compared to HGGs, although a certain overlap between the two groups has been observed [1]. Indeed, despite their high cellularity, HGGs are heterogeneous, presenting necrotic components with higher ADC. In this setting, intravoxel incoherent motion (IVIM) and diffusional kurtosis imaging (DKI) are emerging DWI applications, with the former allowing one to separately estimate tissue diffusivity and microcapillary perfusion, while the latter can analyze non-Gaussian diffusion through high diffusion weighting [4].

For what concerns tumor delineation, malignant glial cells partly extend into peritumoral edema; as a matter of fact, peritumoral edema of glioblastoma multiforme presents a significantly lower minimum ADC (1.149 ± 0.119 × 10^−3^ mm^2^/s) than that of metastases (1.413 ± 0.147 × 10^−3^ mm^2^/s; *p* < 0.05) [5]. The assessment of tumor boundaries using DWI remains challenging; as such neoplastic clusters occupy a small fraction of a voxel. High *b*-values and IVIM, allowing for a detailed study of water diffusion, may help to differentiate tumor from peritumoral edema.

The analysis of DWI changes during therapy has been proposed for a non-invasive and quantitative assessment of the response to treatment. Tissue experiencing a response displays an increase of ADC, while non-responding areas exhibit unaltered values. In the follow-up, recurrences often display similar imaging features to therapy-related changes, such as pseudoprogression or radionecrosis, and perfusion MRI is generally used to distinguish these conditions. For instance, dynamic susceptibility contrast perfusion has shown to be helpful for differentiating tumor recurrence, tumor necrosis, and pseudoprogression in patients treated for cerebral metastases, with relative cerebral blood volume of 2.1 having proven to be the most accurate perfusion parameter [6]. In this setting, the fifth percentile of the ADC histogram at a high *b*-value and mean ADC ≤ 1.3 × 10^−3^ mm^2^/s allowed the differentiation between glioblastoma recurrence and treatment-related changes indicative of pseudoprogression [7].

## 3. Head and Neck

DWI can be useful for head and neck tumor (HNT) characterization, generally applying three b values (*b* = 0, *b* = 500, *b* = 1000). Srinivasan et al. reported significantly (*p* = 0.004) lower mean ADC in malignant HNT (1.071 ± 0.293 × 10^−3^ mm^2^/s) than in benign (1.505 ± 0.487 × 10^−3^ mm^2^/s) [8]. Sakamoto et al. found ADC > 2.10 × 10^−3^ mm^2^/s as a reliable cut-off to identify cysts, with 94% sensitivity, 88% specificity, and 90% accuracy [9]. Notably, there is an overlap of ADC between benign and malignant salivary gland tumors due to very low ADC of Warthin tumors (due to intralesional lymphoid tissue). Habermann et al. reported significantly different (*p* < 0.001) mean ADC between Warthin tumors (0.85 ± 0.1 × 10^−3^ mm^2^/s), mucoepidermoid carcinomas (1.04 ± 0.3 × 10^−3^ mm^2^/s), and pleomorphic adenomas (2.14 ± 0.11 × 10^−3^ mm^2^/s) [10]. DWI has been used also to assess thyroid nodules, with malignant ones presenting much lower ADC. Razek et al. reported significantly lower (*p* = 0.0001) ADC in malignant thyroid nodules (0.73 ± 0.19 × 10^−3^ mm^2^/s) than in benign (1.8 ± 0.27 × 10^−3^ mm^2^/s) [11].

Recent studies have shown promising results regarding the possible application of DWI features as imaging biomarkers of head and neck squamous cell carcinoma (HNSCC) by reporting a correlation with human papilloma virus status, and molecular and histopathological findings of this tumor [12]. Meyer et al. found a correlation between ADC metrics derived from histogram analysis of the ADC maps of HNSCC and p53, Hif1-alpha, VEGF, and Her2-expression, highlighting the potential of DWI in predicting the molecular profile of HNSCC [13]. DWI features have been tested to predict and monitor treatment response of HNSCC. According to most studies, pre-treatment low ADC seems to be a positive predictor in HNSCC, being associated with good local control and treatment response [12]. Regarding response monitoring, several studies have reported an early increase of the ADC of treated HNSCC only a few weeks after treatment commencement, with a significantly higher percentage rise of ADC (>15.5% at 2 weeks, *p* = 0.016) in patients with local control of disease at 2 years [14]. Further, DWI can be helpful in distinguishing residual/recurrent tumor from post-treatment fibrosis/granulation tissue, with the former generally presenting lower ADC (<1.3–1.4 × 10^−3^ mm^2^/s) [12].

**Figure 2:**
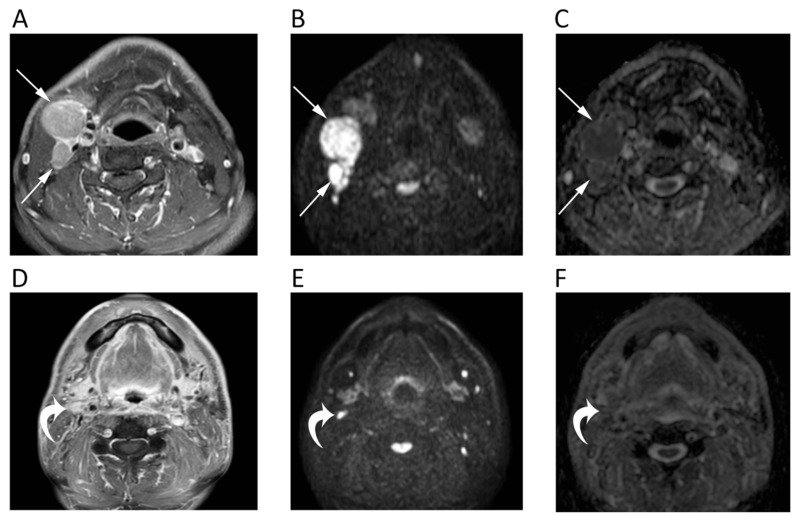
MRI scans of a 65-year-old male patient with oropharyngeal squamous cell carcinoma and lymph nodal metastases. Contrast-enhanced T1w (**A**), high *b*-value DWI (**B**), and corresponding ADC map (**C**) before chemotherapy show round enlarged right cervical lymph nodes (arrows) with restricted pattern of diffusion (mean ADC: 0.891 × 10^−3^ mm^2^/s). The same images after treatment (**D**–**F**) show the good response to chemotherapy with normal size and unrestricted pattern of diffusion of the cervical lymph nodes (curved arrows).

A diagnostic challenge is the differentiation of lymph nodal metastases from both benign nodes and lymphomatous locations, with controversial results reported in the literature. According to most studies, Abdel Razek et al. reported significantly (*p* < 0.04) lower ADC in malignant nodes (metastatic 1.09 ± 0.11 × 10^−3^ mm^2^/s, lymphomatous 0.97 ± 0.27 × 10^−3^ mm^2^/s) than in benign (1.64 ± 0.16 × 10^−3^ mm^2^/s) suggesting a mean ADC of 1.38 × 10^−3^ mm^2^/s as an optimal cut-off with accuracy, sensitivity, and specificity of 96%, 98%, and 88%, respectively (Figure 2) [11].

## 4. Chest

**Figure 3:**
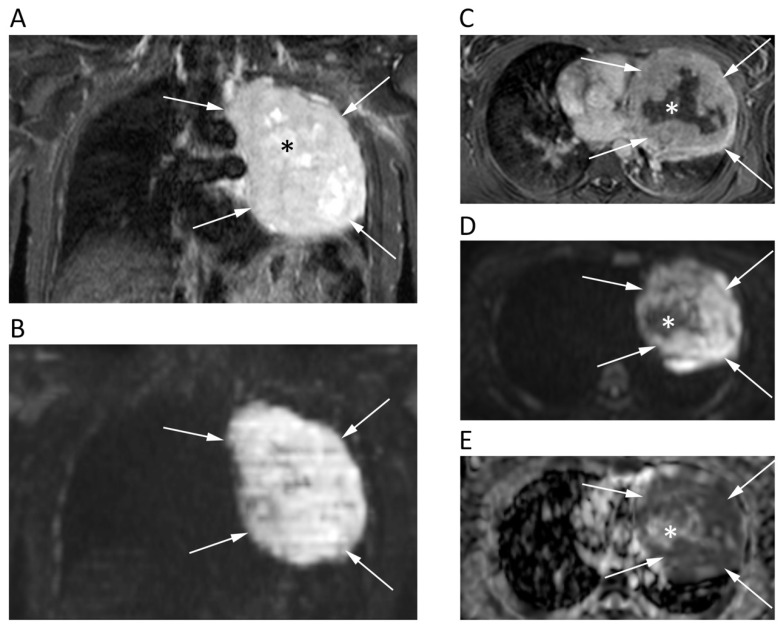
Chest MRI of a 62-year-old male patient with lung carcinoma. The coronal short tau inversion recovery (STIR) (**A**), coronal reconstruction of b 1000 DWI image (**B**), axial post-contrast T1w image (**C**), axial *b* = 1000 DWI image (**D**), and corresponding ADC map (**E**) show a large tumor in the left lung (arrows) with an restricted pattern of diffusion. Note the central necrotic areas (asterisks) presenting fluid signal intensity on STIR (**A**), absent contrast enhancement (**C**) and unrestricted pattern of diffusion (**D**,**E**) with mean ADC of 2.168 × 10^−3^ mm^2^/s. Conversely, the solid component of the tumor shows strong contrast enhancement and very low ADC values (0.818 × 10^−3^ mm^2^/s).

The main issue in chest DWI is that this sequence is highly sensitive to cardiac pulsation and breathing, which can determine a miscalculation of ADC map, and ghost artefacts and blurring, other than susceptibility artefacts that can be encountered at soft tissue and air interfaces [15]. Further, the low proton density of the lungs creates a low signal in all sequences. Nevertheless, Swerkersson et al. demonstrated the high repeatability of whole lesion ADC measurements of lung adenocarcinomas between free breathing and respiratory-triggered DWI, thereby confirming the potential application of this tool in lung tumors [16]. Mori et al. reported similar sensitivities of DWI (70%) and PET (72%) for benign/malignant discrimination of pulmonary nodules, with the former having shown significantly higher specificity (97% vs. 79%; *p* = 0.03) [17]. Çakır et al. reviewed the signal intensity of 48 lung lesions on DWI (*b* = 1000) using a 5-point rank intensity score. When scores were ≥ 3 (signal equal or higher than the spinal cord), lesions were considered as malignant, with this cut-off having reached 93.3% sensitivity and 88.9% specificity [18]. Additionally, DWI has been tested to differentiate necrotic benign and malignant lung lesions using a ratio between ADC of central necrosis and ADC of the wall; that reported better results than measuring the ADC of the wall alone [19]. Authors showed that the wall of malignant lesions had lower ADCs than central necrosis (Figure 3), while benign lesions exhibited lower ADCs in necrotic components [19]. Yang et al. reported similar results of DWI and PET/CT in the differentiation of central bronchogenic tumors from post-obstructive atelectasis, also suggesting the use of ADC maps to guide biopsy and selection of radiotherapy field [20]. Moreover, a new study has reported 100% accuracy of DWI by evaluating ADC changes during the first course of chemotherapy to differentiate chemo-sensitive from chemo-resistant lung cancers early on [21]. Despite these promising results, DWI-MRI has not yet gained space in diagnostic work-up of lung cancers.

On the other hand, there is scarce evidence about the use of DWI in pleural tumors. Coolen et al. tested the DWI to differentiate malignant from benign pleural diseases, and to assess chest wall and diaphragmatic involvement. In their paper, they reported that visual evaluation of pleural pointillism (multiple hyperintense pleural spots on high-*b*-value DWI) could be helpful to distinguish mesothelioma from benign pleural thickening, with even higher diagnostic performance (93% sensitivity, 79% specificity, 88% accuracy) than pleural thickness and shrinking lung [22].

## 5. Breast

**Figure 4:**
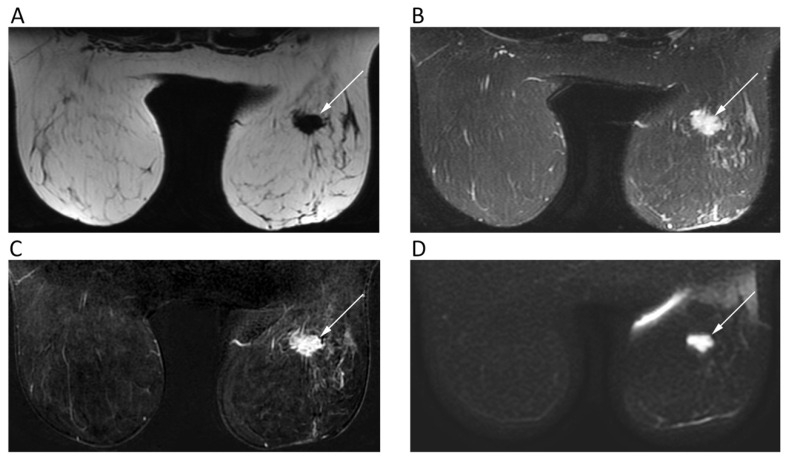
Breast MRI of a 42-year-old female patient with right breast carcinoma. Axial IDEAL fat only (**A**) and IDEAL water only (**B**) images show a right breast carcinoma (arrows) with strong enhancement on post-contrast T1w image (**C**) and high signal intensity on *b* = 900 DWI image (**D**).

Dynamic contrast enhanced (DCE)-MRI has a crucial role in clinical practice of evaluating suspicious breast lesions [23]. However, as an additional tool, DWI has been tested for several purposes, mainly being used with two *b*-values (*b* = 0, *b* = 800). DWI has been investigated as a stand-alone approach for breast cancer detection, although the diagnostic performance of DCE-MRI still exceeds that of DWI, with the latter presenting 84%–86% sensitivity and 76%–79% specificity [23]. Nevertheless, the added value of DWI is proven by the higher accuracy of combined DCE/DWI-MRI (94%) than that of DCE-MRI alone (85%) for breast cancer detection [24]. Further, although breast malignancies present a restricted pattern of diffusion (Figure 4), DWI has a limited role in distinguishing benign from malignant breast lesions if compared to DCE-MRI. Moreover, invasive tumors tend to present lower ADC than carcinoma in situ [25]. In this setting, a high *b*-value (*b* = 900/1000) improves the discrimination of breast lesions from the neighboring soft tissue [26], although the best choice of *b*-values to be used in breast DWI is still debated. In fact, higher b values increase the specificity of DWI sequence at cost of decreased signal-to-noise ratio, thereby leading most authors to prefer a *b* = 800 image [27].

DWI also proved to be helpful for pre-operative work-ups of multifocal–multicentric breast carcinoma to correctly assess tumor size [28]. Regarding its prognostic role, the ADC of breast cancer has been associated with many biological markers of disease. A low ADC is associated with positive expression of estrogen receptors, increased microvascular density, and increased Ki-67, although the correlation between prognostic parameters and ADC is still discussed [29]. Further, concerning treatment response, ADC has been reported to increase earlier compared to the occurrence of morphological changes [26]. Notably, breast DWI may lead to false positives due to hematomas, highly-cellulated fibroepithelial lesions, abscesses, cysts with thick content, and intra-mammary lymph nodes. On the other hand, mucinous carcinoma and small lesions can lead to false negatives [26].

## 6. Hepatobiliary

The role of DWI in liver imaging has been extensively investigated. The most used DWI sequence for liver imaging, nowadays included in routine liver protocol, is single-shot spin-echo fat-suppressed echo-planar imaging with at least three or four *b*-values, generally with the highest being *b* = 600. Compared to T2-weighted, DWI is superior in the diagnostic work-up of focal liver lesions, showing better performance in terms of detection rate and sensitivity even for small lesions (1–3 cm), which may be otherwise missed or misinterpreted [30]. DWI is considered reliable for detection and monitoring of liver metastases, especially those from neuroendocrine tumors, which are highly-cellulated, thereby being easily recognized in DWI [31].

**Figure 5:**
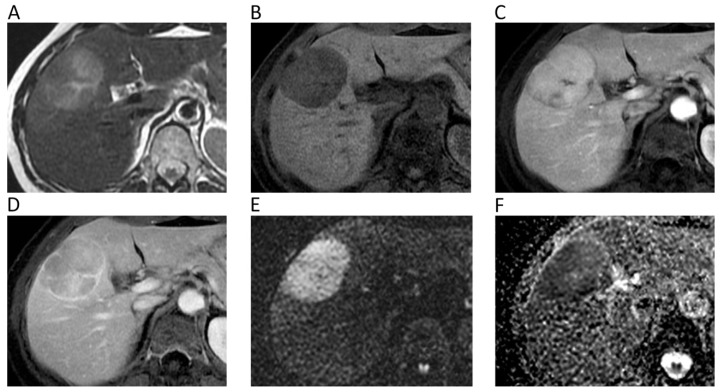
Liver MRI of a 54-year-old female patient with histologically proven hepatocellular carcinoma (HCC) on her healthy liver. Axial T2w (**A**) and pre-contrast fat-suppressed 3D-gradient-echo T1w (**B**) images show a large mass with strong contrast enhancement on the arterial phase (**C**), wash-out and rim enhancement on the portal phase (**D**), high signal intensity on *b* = 800 DWI image (**E**), and low ADC values (mean ADC: 1.018 × 10^−3^ mm^2^/s) on the corresponding ADC map (**F**).

ADCs from 1.5 to 1.6 × 10^−3^ mm^2^/s have been reported as cut-offs to identify malignant liver lesions [32]. However, a consensus is still lacking about the optimal ADC cut-off due to overlap between benign and malignant ones. As a general rule, signal hyperintensity on higher *b*-value DWIs with low ADCs are associated to malignant highly-cellulated and inflammatory (i.e., abscesses) lesions due to diffusion restriction (Figure 5) [32].

Currently, DWI is not included in the European Association for the Study of the Liver non-invasive diagnostic criteria for hepatocellular carcinoma (HCC). However, it has been shown that DWI increases sensitivity and positive predictive value (PPV) for the detection of HCCs < 20 mm thanks to better contrast-to-noise ratio and background suppression of normal parenchyma [33]. In addition, DWI has a promising role in anticipating HCC grading and distinguishing poorly from well/moderately differentiated HCC [34]. Furthermore, indeterminate hypovascular lesions with diffusion restriction in cirrhosis are often prone to develop hypervascularity and overt imaging features of HCC, making DWI a strong predictor for progression to hypervascular HCC [35]. A typical DWI finding of cholangiocarcinoma is the target sign, which is encountered in the hepatobiliary phase too. This imaging feature can be helpful to differentiate cholangiocarcinoma from HCC, being observed in about 87% and 15% of cases, respectively [36]. DWI can be an additional tool also to differentiate benign and malignant gallbladder lesions. Specifically, a recent meta-analysis including 557 patients has shown higher accuracy of qualitative assessment when compared with quantitative analysis of DW images (94% vs. 88%), especially when using 3T and thinner slices (≤5 mm) [37].

Regarding prediction of treatment response, liver metastasis with high ADC seem to be associated with poor response to chemotherapy [38]. DWI can help also in differentiating non-tumoral tissue from local tumor relapse after treatment. Non-responding tumors do not present significant ADC changes after treatment, while ADC significantly increases in responding ones [38]. As for loco-regional treatments, Schraml et al. found significantly lower ADC in tumoral viable tissue after radiofrequency ablation than in the ablation zone and normal parenchyma (1.02 × 10^−3^ mm^2^/s vs. 1.31 × 10^−3^ mm^2^/s) [39].

## 7. Pancreas

**Figure 6:**
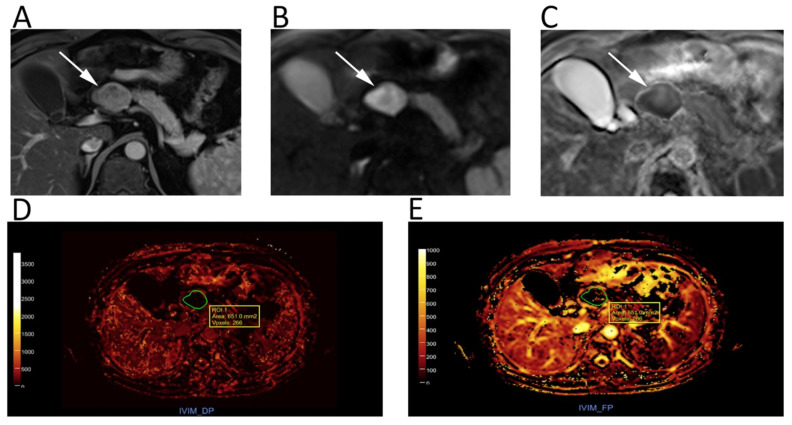
Upper abdomen MRI of a patient with G2 pancreatic neuroendocrine neoplasm. A heterogeneous hypervascular lesion (arrows) with well-defined margins can be seen on the axial arterial phase image (**A**). The lesion is clearly hyperintense on *b* = 800 DWI (**B**), with low ADC values on the corresponding ADC map (**C**). IVIM maps show that the tumor has low values of fast diffusion (**D**) Dp, but relatively preserved perfusion fraction (**E**) Fp, consistent with a neuroendocrine neoplasm.

DWI is widely adopted as a part of pancreas MRI protocol. Pancreatic tumors, even if small, almost invariably show diffusion restriction, presenting as focal hyperintense areas on high-*b*-value DWI with corresponding low ADC. Previous studies reported high diagnostic performance for identification of pancreatic ductal adenocarcinoma using DWI, with reported accuracy, sensitivity, and specificity of 96%, 96%, and 99%, respectively [40,41]. Similarly, Kartalis et al. found very high diagnostic performance of DWI (92% sensitivity, 97% specificity, 96% accuracy) to differentiate malignant (mean ADC 1.4 ± 0.3 × 10^−3^ mm^2^/s) from benign (2.57 ± 1.17 × 10^−3^ mm^2^/s) pancreatic lesions [42]. Notably, the DWI appearance of PDAC can be very similar to that of autoimmune pancreatitis, which presents as a focal pancreatic enlargement with even lower ADC, due to fibrosis and inflammatory cell infiltration [40,41]. DWI has been also found to improve the detection of pancreatic neuroendocrine neoplasms (pNENs) [40]. Nevertheless, concurrent chronic pancreatitis may represent a confusing factor for the identification of pancreatic lesions [41]. Further, the differentiation of benign and malignant pancreatic lesions based only on ADC quantification is not so straightforward because of a wide overlap of ADC values [40,41]. Controversial results were also found to differentiate cystic lesions by means of DWI alone [40]. More consistent results have been described by using IVIM-DWI (Figure 6): perfusion-related parameters (i.e., perfusion fraction and fast component of diffusion) showed excellent inter-observer agreement and accuracy for identifying solid pancreatic lesions, and distinguishing malignant from benign intraductal papillary mucinous neoplasms [40,41].

Prognosis in patients with pancreatic tumors is highly influenced by tumor stage and grade. Several studies demonstrated that DWI is a reliable tool to identify liver metastases from pancreatic tumors [41] and to predict the aggressiveness of such lesions, as ADC seems to be lower in patients with worse clinical course and prognosis [41]. Regarding pNENs, a recent meta-analysis reported that ADC had a pooled 84% sensitivity and 87% specificity for distinguishing G1 from G2/3 pNENs and 93% and 92% in distinguishing G1/2 from G3, respectively [43]. A very limited number of studies focused on post-treatment DWI assessment of pancreatic lesions, but this topic deserves further investigations. Indeed, promising results have been recently reported by a preliminary study that investigated the role of MRI to assess the response of pancreatic ductal adenocarcinoma xenografts to iodine-125 seeds brachytherapy in mice [44]. The mean ADC significantly increased (*p* < 0.05) 14 and 60 days after treatment and was linearly proportional to the mean apoptotic cell density (*p* = 0.015; Spearman’s coefficient = 0.945).

## 8. Esophago-Gastro-Intestinal

Endoscopic ultrasonography is the gold standard for local staging of esophageal cancer, while CT and PET/CT are used for whole-body staging. Few studies have tested the diagnostic performance of DWI showing higher specificity (83% vs. 67%) but lower sensitivity (67% vs. 100%), of DWI when compared with endoscopic ultrasonography [45]. DWI has also shown good accuracy for nodal staging (66%), with 100%sensitivity as endoscopic ultrasonography [45]. Nevertheless, there is still no enough evidence to introduce DWI in pre-operative work-up of esophageal cancer.

Gastric cancer is highly-cellulated and shows significantly lower ADC (1.115 ± 0.156 × 10^−3^ mm^2^/s) than normal gastric wall (1.621 ± 0.292 × 10^−3^ mm^2^/s) or benign diseases (1.790 ± 0.359 × 10^−3^ mm^2^/s) [46]. Although ADC seems to be an independent prognostic factor in gastric cancer with interesting correlations with histologic grading, subtype, and Ki67 index, this sequence is not routinely used in patients with gastric cancer [47]. Indeed, the imaging quality of DWI is affected by susceptibility artifacts, gas, and gastric movements that lead to image distortion and ghosting. However, Cai et al. have recently proposed a reduced field-of-view DWI to yield better image quality and more accurate ADC measurements [48]. Further, Further, DWI has proven to be accurate in detecting nodal locations of gastric cancer in pre-operative staging (sensitivity, specificity, and accuracy of 79%, 69%, and 81%, respectively), being even superior to PET/CT [49].

Although CT is the imaging modality of choice for the preoperative assessment of colon cancer, DWI-MRI has been increasingly tested as an alternative method to provide quantitative characterization of the tumor before, during, and after chemotherapy [50]. Different studies demonstrated the good performance of DWI-MRI in local staging of colon cancer, including bowel wall invasion, vascular infiltration, and serosal involvement [51,52]. Dam et al. investigated the diagnostic performance of unenhanced MRI with combined T2-weighted and DWI to stage sigmoid-colon cancer, reporting mean ADC values always lower than 1.000 × 10^−3^ mm^2^/s and accuracies of 89%–94%, 60%–66%, and 60%–77% in staging early versus advanced cancer, N-stage, and vascular invasion, respectively [50]. On the other hand, the authors were cautious about these results, as these were limited to the sigmoid colon, which is less affected by motion artifacts in comparison with transverse and right colon.

**Figure 7:**
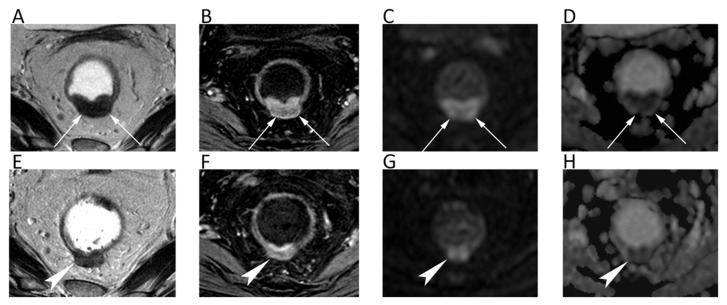
MRI scans of a 66-year-old female patient with rectal cancer at the initial diagnosis and two months later after neoadjuvant chemotherapy. Axial T2w image (**A**) and axial post-contrast fat-suppressed 3D GRE T1w image (**B**) show the tumor along the posterior rectal wall (arrows) with high signal intensity on the axial *b* = 600 DWI image (**C**) and low ADC (mean ADC: 0.651 × 10^−3^ mm^2^/s) on the corresponding ADC map (**D**). Post-treatment axial T2w image (**E**) shows the reduction in size of the lesion (arrowheads) with decrease of contrast enhancement (**F**) and slight increase of ADC values (**G**,**H**).

An MRI is part of both primary staging and restaging of rectal cancer, allowing one to stratify treatment by identifying those patients at risk of local recurrence. DWI can be helpful in primary staging of rectal cancer in adjunction to conventional sequences, applying at least two b values and with *b* = 1000 generally being used as the highest *b*-value. A recent study reported an improved specificity (63.2% to 75.9%) of MRI in detecting perirectal infiltration by adding DWI to T2-weighted [53]. According to the recent international guidelines, DWI should be included in MRI protocol, especially for restaging rectal cancer to differentiate complete from partial response, with still no consensus on the added value of DWI in the assessment of primary T and N-staging [54]. Indeed, a meta-analysis on the restaging of rectal cancer of 1556 patients after chemo-radiotherapy showed the high sensitivity of DWI (83.6%), with an improvement of the negative likelihood ratio [55]. Further, previous studies have shown the added value of T2-weighted and DWI findings combined with endoscopy, leading to a post-test probability of 98% for predicting complete response after chemo-radiotherapy [56]. Nevertheless, DWI alone is not accurate enough in restaging rectal cancer; thus, the conjunction with standard sequences is recommended. Notably, although the evaluation of ADC changes during treatment has shown to be promising to assess treatment response (Figure 7), DWI should be only qualitatively assessed, with ADC quantification having still no role in clinical practice due to a lack of protocol standardization and agreed cut-off values [54].

## 9. Gynecological

DWI in the field of gynecology is usually acquired in axial plane (short axis of the organ for uterus and cervix), angled similarly to T2-weighted, in order to co-register them and optimizing anatomic information [57]. As for rectal cancer, two b values are generally used with *b* = 1000 as the highest.

MRI is adopted for endometrial cancer detection, even though small cancers may not be detected by conventional protocol. ADCs of endometrial cancers are significantly lower than those of normal endometrium, with high-grade cancers presenting even lower ADCs than lower-grade ones [57]. Combined DWI and T2-weighted images are more accurate than T2-weighted alone in the assessment of myometrial invasion with a sensitivity of up to 92%, which is a key pre-operative feature to decide the surgical approach (type of hysterectomy and lymph node dissection) [58]. DWI can be also useful in distinguishing benign lesions, such as degenerated leyomioma and adenomyosis, from malignant myometrial lesions with similar T2-signals [57].

**Figure 8:**
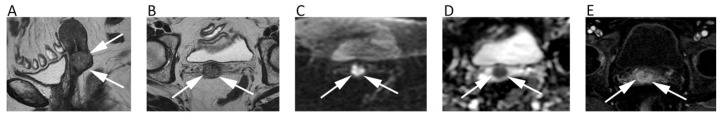
A cervical cancer without parametrial invasion in a 70-year-old woman. The lesion (arrows) is slightly hyperintense compared to normal cervical stroma on sagittal (**A**) and axial (**B**) T2w, whereas it is highly hyperintense on *b* = 800 DWI (**C**) and hypointense on the corresponding ADC map (**D**), with moderate contrast enhancement on fat-suppressed 3D-GRE T1w (**E**).

Regarding cervical cancer, a lesion that is visible with a conventional MRI indicates a stage IB or higher, with the tumor usually being well visible on T2-weighted. However, DWI has been shown to improve small cancers’ detection after biopsy, since post-procedure inflammatory changes may alter the normal anatomy [58]. DWI is also more accurate than T2-weighted at assessing tumor size and improves the accuracy in detecting parametrial invasion (tumors with parametrial invasion have significantly lower ADCs than those without (*p* = 0.034) [59] and peritoneal spread of disease (90% sensitivity, 95.5% specificity) (Figure 8) [58]. When cervical cancer is not eligible for surgery, ADC is a useful biomarker for predicting response to chemo-radiotherapy [57]. Studies have shown that a low pre-treatment ADC is a predictor of good response and an early ADC increase, even before the reduction of tumor size [57].

DWI seems also to increase the diagnostic performance of MRI in the diagnosis of ovarian cancer when it is added to conventional sequences [60]. A cut-off ADC of 1.15 × 10^−3^ mm^2^/s allows one to distinguish benign from malignant/borderline malignant lesions with 74% sensitivity and 80% specificity [61]. Further, the presence of a solid component within an adnexal mass that is hypointense both on T2-weighted and high-*b*-value DWI is highly specific for benignity, while a solid component with intermediate T2 signal and high b1000-DWI signal is associated with a positive likelihood ratio of 4.5 for a malignant adnexal tumor [62]. However, care must be taken in evaluating lesions with high fluid content, such as tumors with cystic/necrotic areas and mucinous tumors [58].

Regarding the evaluation of loco-regional lymph nodes, in gynecological malignancies there is considerable overlap in ADC of metastatic and non-metastatic lymph nodes; therefore, the role of DWI in this field is still controversial [61]. For what concerns peritoneal implants of ovarian cancer, the lesions show restricted diffusion and DWI can be helpful to quickly identify peritoneal locations of disease, although small implants (<5 mm) could be missed due to susceptibility artifacts at air-tissue interfaces, such as lesions adherent to the bowel and diaphragmatic surface [57].

## 10. Urinary System and Adrenal

Several studies have investigated the diagnostic performance of DWI for renal mass characterization [63]. DWI has shown to be an additional tool to differentiate malignant from benign cysts, with ADC progressively decreasing with the increase of Bosniak category [64]. In a meta-analysis on the characterization of renal masses, Kang et al. reported moderate accuracy of DWI (78% specificity, 86% sensitivity) to identify renal malignancies [65]. The authors also highlighted the unclear performance for ascertaining clear cell renal cell carcinoma (ccRCC) histologic grade [65], a finding that was confirmed by Woo et al. that reported moderate accuracy (86% specificity, 78% sensitivity) of DWI to differentiate low-grade and high-grade ccRCC [66]. Recently, Ding et al. have reported excellent diagnostic accuracy (89%) of IVIM in the differentiation between ccRCC and benign renal tumors, especially when evaluating true diffusivity, while DWI and DKI demonstrated similar performance [67]. Ding et al. reported significantly higher ADC values of ccRCCs (1.78 ± 0.29 × 10^−3^ mm^2^/s) than those of non-RCC (1.31 ± 0.34 × 10^−3^ mm^2^/s) and benign renal tumors (1.35 ± 0.29 × 10^−3^ mm^2^/s) [67]. Previous studies have tried to use DWI to identify the different RCC subtypes and to discriminate ccRCC from oncocytoma [63]. Although ccRCC tends to display higher ADC than papillary and chromofobe RCCs, these values are not so different and present an overlap like that of ccRCC and oncocytoma, which are often not distinguishable [63]. Aslan et al. have proposed to use DWI to differentiate Wilms tumors from neuroblastomas, which are the most common pediatric abdominal malignancies [68]. In their study, two observers found a significantly higher mean ADC for Wilms tumors (0.768–0.787 × 10^−3^ mm^2^/s) than for neuroblastomas (0.525–0.529 × 10^−3^ mm^2^/s; *p* ≤ 0.011) [68]. Further, Goyal et al. recently published promising data about MRI texture analysis in subtyping and grading of RCC, including DWI, with several texture parameters having showed high diagnostic performance (accuracy > 80%) [69].

Multi-parametric MRI (mpMRI) including T2-weighted, DWI, and DCE has emerged as an optimal diagnostic tool for local staging of bladder cancer. Takeuchi et al. described the DWI features of bladder cancer according to local stage: ≤T1, flat tumor with thickened submucosa or a submucosal stalk without restricted diffusion; T2, non-flat tumors that bulge with smooth surfaces toward the muscle and those without a submucosal component; T3, irregular tumor margin toward the peri-vesical fat; T4, irregular tumor margin extending into a neighboring organ, or pelvic or abdominal wall [70]. Notably, DWI can be used to estimate tumor grade: low-grade bladder cancer presents a significantly higher ADC than high-grade [71]. Further, Theony and colleagues proposed identifying normal-sized pelvic nodal metastases from bladder cancer as lymph nodes with higher signals than inguinal nodes on high-*b*-value DWI in addition to traditional morphologic criteria, reaching sensitivity of 64%–79% and specificity of 79%–85% [72]. Moreover, DWI might be helpful also to image ureteral cancer. When compared to conventional sequences, qualitative analysis of DW images increases both sensitivity (91%–94% vs. 72%–75%) and accuracy (77%–90% vs. 66%–67%), with *b* = 1500 DW images improving specificity even more than *b* = 500 images (84%–87% vs. 66%–68%) [73]. On the other hand, there is scarce evidence regarding the possible application of quantitative DWI analysis through ADC measurements of ureteral tumors. In this setting, initial good results have been reported by Roy et al., who found an optimal cut-off value of 1.100 × 10^−3^ mm^2^/s for predicting benignancy, with the sensitivity, specificity, PPV, and negative predictive value (NPV) of 90.9%, 82.6%, 83.3%, and 90.5%, respectively [74].

**Figure 9:**
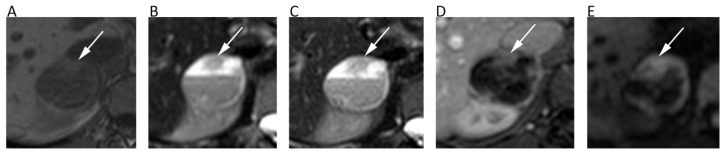
A 28-year-old male patient with pheochromocytoma of the right adrenal gland. Axial in-phase T1w (**A**), axial T2w (**B**), and axial fat-suppressed T2w (**C**) images show a right adrenal cystic lesion with fluid-fluid level. Axial post-contrast fat-suppressed 3D GRE T1w (**D**) and *b* = 600 DWI (**E**) allow one to better depict the solid component of the lesion consisting of the peripheral wall and the anterior nodular portion (arrows), with no restricted pattern of diffusion in central cystic part.

Similarly to renal lesions, adrenal masses are often incidentally identified by abdominal imaging examinations, with non-hyperfunctioning adenomas representing the most common lesion [75]. Contrast-enhanced CT and chemical-shift MRI are generally used as helpful tools to characterize adrenal lesions [76]. Several authors tried to understand the role of DWI for differentiating benign and malignant adrenal masses with poor results. Miller et al. reviewed 160 adrenal masses reporting that ADC was not helpful for distinguishing benign (1.67 × 10^−3^ mm^2^/s) from malignant lesions (1.61 × 10^−3^ mm^2^/s; *p* > 0.05) [77]. Further, in their series lipid-rich and lipid-poor adenoma did not show significantly different ADCs (*p* > 0.05) [77]. Similar results were achieved by Sandrasegaran et al. who stated that DWI has limited usefulness for characterizing adrenal lesions [78]. Notably, a study performed with a 3T MRI scanner on 40 patients showed that malignant pheochromocytomas had significantly higher ADC than benign ones (1.175 ± 0.132 × 10^−3^ mm^2^/s vs. 0.918 ± 0.124 × 10^−3^ mm^2^/s; *p* < 0.001) (Figure 9) [79]. Thus, DWI has still a marginal role to characterize renal and adrenal masses.

## 11. Prostate

**Figure 10:**
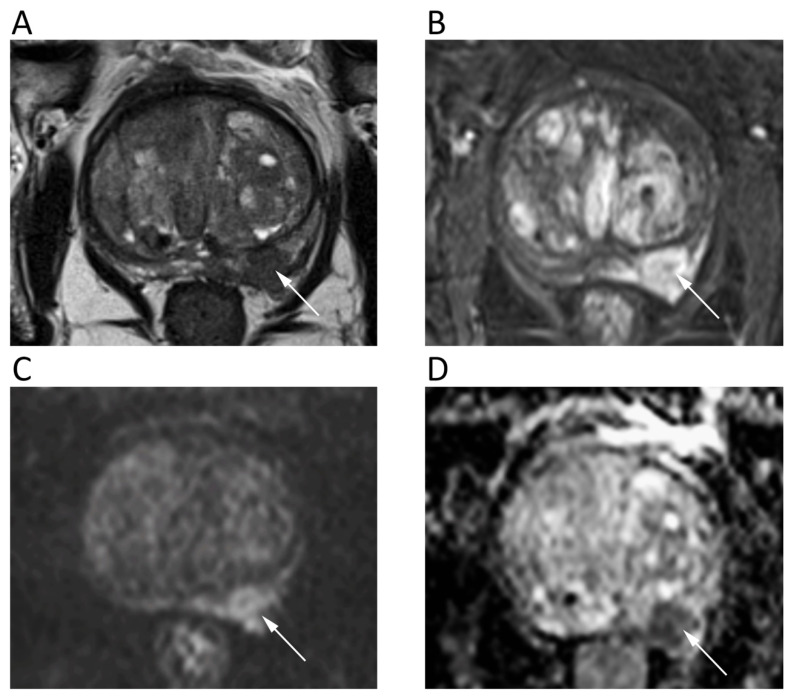
A 68-year-old male patient with prostate cancer. Axial T2w (**A**) and post-contrast fat-suppressed T1w (**B**) images show a hypointense nodule in the left peripheral zone (arrows) with high signal intensity on *b* = 1000 DWI image (**C**) and restricted pattern of diffusion on the corresponding ADC map (**D**).

The mpMRI is widely used to detect, localize, and stage prostate cancer (PCa). The prostate imaging reporting and data system (PI-RADS v2.1) for mpMRI is the most common method for standard reporting. It consists of a 5-point scale based on the likelihood that a combination of mpMRI findings on T2-weighted, DWI, and DCE correlates with the presence of a clinically significant PCa [80]. To increase the accuracy of DWI, both ADC map and high-*b*-value DWI images should be evaluated, generally applying three b values, with the lowest *b* = 0 and the highest *b* = 1400/1500, although some authors suggest using *b* = 2000. Nevertheless, there is no widely accepted optimal high-*b*-value in the literature. Imaging with higher *b*-values suppresses healthy background tissue, highlighting PCa tissues with restricted diffusion and lower ADC (Figure 10). Further, it should be considered that transitional zone and peripheral zone tumors may present different perfusion and diffusion parameters [81].

Although ADC has shown to inversely correlate with histologic grades, there is considerable overlap between benign prostatic hyperplasia, low-grade PCa, and high-grade PCa. Qualitative visual assessment of DWI is often used as the primary method. An ADC threshold of 750–900 mm^2^/s has been proposed to ease the differentiation between benign and malignant prostate tissues, with ADC below it having been correlated with clinically significant PCa [80]. Authors have identified a moderate correlation between ADC and Gleason score of PCa in the peripheral zone and weak correlation in the transitional zone (pooled correlation coefficient of 0.48 and 0.22, respectively) [82], although better results have been reached using the ADC ratio as a more accurate and reproducible method [83]. Previous studies reported similar (*p* = 0.83) diagnostic performance of biparametric MRI without DCE (pooled sensitivity and specificity of 0.74 and 0.90, respectively) and mpMRI (pooled sensitivity and specificity of 0.76 and 0.89, respectively) for the diagnosis of PCa, suggesting the former as a valuable first-line imaging test due to its robust sensitivity [84]. Notably, Gong et al. have recently reported the high diagnostic performance of radiomic models based on biparametric MRI to non-invasively identify high-grade PCa, with accuracy ranging from 0.787 and 0.801 [85]. However, despite the limited role of DCE in determining the overall PI-RADS category, in some instances DCE may assist in the detection of PCa and the PI-RADS Committee suggests to reserve biparametric MRI only for selected clinical scenarios.

## 12. Lymph Nodes and Spleen

**Figure 11:**
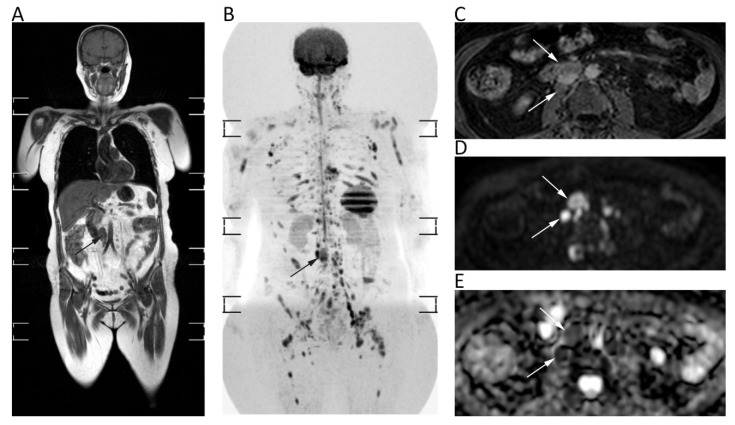
Whole-body MRI of a 58-year-old female patient with non-Hodgkin lymphoma. Whole-body T1w (**A**) and coronal MIP grey-scale inverted DWI (**B**) show multiple nodal and bone locations of disease. Note the periaortic lymph nodal locations (arrows) with moderate contrast enhancement on axial fat-suppressed 3D GRE T1w (**C**) and restricted pattern of diffusion on axial *b* = 800 DWI (**D**) and corresponding ADC map (**E**, mean ADC: 0.761 × 10^−3^ mm^2^/s).

Whole-body (WB)-DWI-MRI has emerged as an accurate tool to stage lymphoma using DWI with background suppression (DWIBS). DWIBS is a single-shot echo-planar sequence characterized by signal acquisition with sensitivity encoding to improve resolution and decrease artefact at air interfaces, free breathing acquisition to increase signal to noise ratio, and homogeneous fat suppression by STIR technique [15]. DWI enables one to detect lymphomatous locations as lesions with very high signals on high-*b*-value images—generally higher signals than those of the spinal cord and muscles—and low ADCs due to the high cellularity and elevated nuclear-to-cytoplasm ratio of lymphoma [15]. Nevertheless, there is still no consensus on the *b*-values to be used on DWI sequences, although at least two *b*-values are applied with *b* = 1000 as the highest *b*-value image. The lack of consensus concerns also the best ADC cut-off to differentiate normal from lymphomatous lymph nodes, although several studies have shown that the latter often present mean ADC lower than 0.8 × 10^−3^ mm^2^/s (Figure 11) [86,87].

Moreover, it is not clear whether mean or minimum ADC should be used. In lymphoma staging, WB-DWI-MRI showed similar diagnostic performance to FDG-PET/CT with excellent agreement (k = 0.88) and high sensitivity (98%) and specificity (97%) for nodal involvement, presenting even higher accuracy than CT for both nodal (accuracy of WB-DWI-MRI and CT: 95.5% and 94.3%) and extra-nodal disease (accuracy of WB-DWI-MRI and CT: 98.5% and 76.3%) [87,88]. Despite the promising results of the above-mentioned studies, there is still no clinical application of ADC measurements for lymphoma.

In addition to its proven accuracy in lymphoma staging, WB-DWI-MRI has shown promising results to assess response after treatment. In most studies a qualitative evaluation of DWI and ADC maps has been used to evaluate treatment response with excellent results (94.7% sensitivity, 99.3% specificity). However, there is no enough evidence to introduce quantitative evaluation of treatment response by DWI in clinical practice, with only one study having proposed a mean ADC cut-off of 1.2 × 10^−3^ mm^2^/s to differentiate responding from non-responding lesions [89].

Imaging characterization of splenic lesions has always been considered challenging with morphologic sequences, with helpful information provided by contrast enhancement behavior. Indeed, hypervascular enhancement pattern is typical of benign lesions [90]. Splenic parenchyma has a spontaneous restricted diffusion, potentially leading to an underestimation of DWI appearance of the lesions. Nevertheless, DWI combined with conventional sequences proved to be useful in the differentiation between malignant and benign splenic lesions, with the former presenting mean ADC of 0.73 × 10^−3^ mm^2^/s and the latter 1.21 × 10^−3^ mm^2^/s [90]. WB-MRI-DWI has been reported as an accurate tool to also evaluate splenic involvement in lymphoma when compared with FDG-PET/CT, allowing one to detect the splenic location of disease with sensitivity, specificity, PPV, and NPV of 85.7%, 96.5%, 85.7%, and 96.5%, respectively [91].

## 13. Bone Tumors

The use of DWI for bone tumors (BTs) has been advocated for several scenarios, including the differentiation of benign from malignant tumors or vertebral fractures, and the therapeutic follow-up evaluation [92,93].

The first thing to consider is that ADCs of BTs (regardless the type, whether benign or malignant) are usually higher than those of normal yellow marrow, since any condition that alters or replaces the normal microarchitecture of bony trabeculae and fatty marrow results in increased free water movement, with higher ADC compared to adjacent marrow. Normal yellow marrow ADC in adults has been reported to be low (0.3–0.4 × 10^−3^ mm^2^/s), while children usually have a higher ADC for normal bone marrow [94].

**Figure 12:**
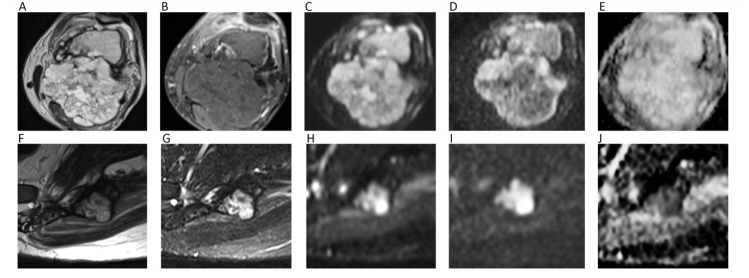
The left femur G3 chondrosarcoma (**A**–**E**) of a 76-year-old male patient. Axial T2w image (**A**) shows a hyperintense bone lesion with a large posterior soft tissue mass presenting poor contrast enhancement on fat-suppressed T1w image (**B**). The lesion shows high signal on *b* = 0 DWI image (**C**), decreased signal on *b* = 1000 DWI image (**D**), and unrestricted pattern of diffusion on the corresponding ADC map (**E**, mean ADC: 2.303 × 10^−3^ mm^2^/s). The Ewing sarcoma (**F**–**J**) of the left iliac bone of a 31-year-old male patient. The tumor presents as an esofitic bone lesion with intermediate signal on axial T2w image (**F**) and high signal on axial STIR image (**G**). The lesion shows intermediate-high signal on *b* = 0 DWI image (**H**), higher signal on *b* = 1000 DWI image (**I**), and restricted pattern of diffusion on the corresponding ADC map (**J**, mean ADC: 0.899 × 10^−3^ mm^2^/s).

As a general rule, malignant BTs show lower ADC compared to benign BTs [93]. However, it has been reported that certain types of benign BT may present low ADC (<1.0 × 10^−3^ mm^2^/s), such in the case of non-ossifying fibroma or giant cell tumor, probably due the condensed collage fibers of the former and the elevated cellularity of the latter [95]. Thus, DWI should not be used alone, but as a supplementary tool together with conventional MRI sequences. ADC is generally low in osteosarcoma, despite certain variability related to the different histological characteristics, with the solid cellular tumor component showing lower ADC compared to necrotic parts [96]. The use of ADC in chondrosarcomas is controversial, with DWI having a limited role in differentiating between benign and malignant cartilage tumors, due to the fact that tumor cells are more exiguous within the myxoid matrix [93]. Indeed, Douis et al. showed that ADC did not significantly differ between low-grade and high-grade chondrosarcomas (Figure 12A–E) [97].

With regard to spine, it has been shown that metastasis and malignant spinal BT generally show lower ADC compared to benign lesions [98]. When considering only primary spinal BTs, it has to be taken into account that certain overlap may exist for ADC values, although primary hypercellular malignant spinal BTs (such as Ewing’s sarcoma, lymphomas, or multiple myelomas) usually show very low ADC (<1.0 × 10^−3^ mm^2^/s) (Figure 12F–J) [93,98]. When differentiating benign from malignant vertebral fractures, it has to be considered that literature usually refers to benign fractures as those that arise from osteoporosis and not from benign tumors. In such cases, benign fractures usually show higher ADCs [99]. A recent meta-analysis by Luo et al. confirmed the utility of ADC, suggesting one to use a threshold of mean ADC < 1.4 × 10^−3^ mm^2^/s to reasonably detect malignancy [99].

Response to neoadjuvant chemotherapy in terms of tumor necrosis is an important prognostic factor. Although it has not yet standardized in clinical practice, DWI can be useful for evaluating patients before and after chemotherapy, with several papers reporting an increase in both minimum and mean ADC during follow-up [100]. This is supposed to be related to the different ADCs of necrotic areas compared to viable tumors; this difference may not be detected on conventional T2-weighted images, in which tumor and necrosis could be both hyperintense. The use of ADC for therapeutic response has also been investigated in patients with bone metastases and myeloma. In general, ADC values increased after therapy, confirming the utility of DWI as a biomarker also in such conditions [101,102].

## 14. Soft Tissue Tumors

**Figure 13:**
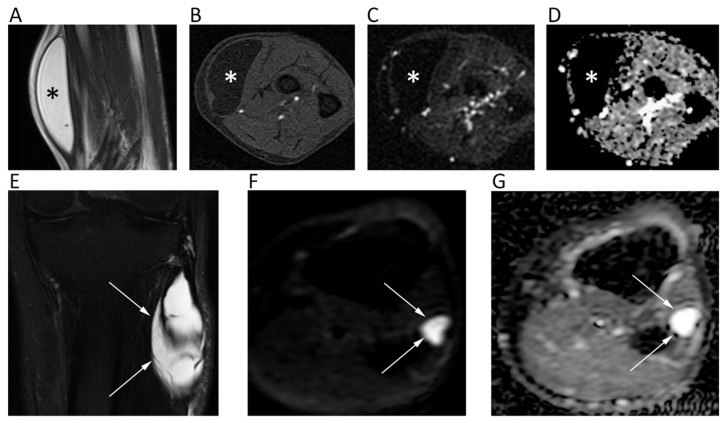
MR images of an intramuscular lipoma of the forearm (**A**–**D**) and a perineural ganglion cyst of the leg (**E**–**G**) of two different patients. The lipoma (asterisk) shows a homogeneous high signal on coronal T2w (**A**) and a low signal on axial fat-suppressed T1w (B), *b* = 800 DWI (**C**); the corresponding ADC map (**D**). Note the absence of diffusion restriction and the intrinsically low ADC values of this benign lipomatous lesion. The ganglion cyst (arrows) displays high signal intensity on coronal fat-suppressed T2w (**E**), axial *b* = 800 DWI (**F**), and axial ADC map (**G**). High *b*-value DWI shows high signal due to T2 shine-through effect; the ADC map confirms the absence of diffusion restriction and the cystic nature of the lesion (ADC: 2.603 × 10^−3^ mm^2^/s).

DWI in soft tissue tumors (STTs) is generally used with at least three *b*-values (from *b* = 50 to *b* = 1000) [103,104]. According to some authors, minimum ADC may be more accurate for characterization compared to mean ADC, while mean ADC should be preferred for tumor follow-up and treatment response evaluation [105]. Tissue cellularity, however, is not the only feature influencing diffusion. In vascular STTs, ADC increases due to tissue perfusion and intravascular water component, leading to possible overlapping between benign and malignant lesions [103]. To overcome this limitation, perfusion-insensitive ADC values can be used. Myxoid STTs show higher intrinsic diffusion coefficients due to their elevated mucin contents, and the difference in ADC between benign and malignant myxoid STTs was reported to be not statistically significant in some studies [103,104]. Similarly, both benign and malignant fatty STTs have very low ADC [106] (Figure 13A–D). Concerning tumor characterization, several studies reported significantly lower ADC of malignant STTs than benign ones, although a variable degree of overlap has been always reported [104,107,108]. According to a recent study by Choi et al., this overlap seems to be especially due to the high ADCs of myxoid malignant STTs and the low ADCs of giant cell tumors, fibromas, and schwannomas [107]. ADCs greater than 2.5 × 10^−3^ mm^2^/s were reported to yield high NPVs for ruling out malignant lesions (100% specificity) (Figure 13E–G) [108]. Oka et al. reported the use of DWI for the differential diagnosis between chronic hematomas and haemorrhagic malignant STTs, reporting significantly higher ADC in hematomas without any overlap [109].

Regarding the evaluation of response after chemo-radiotherapy, the histological response depends on the degree of necrosis, which correlates with enhancement behavior, although responding STT can present treatment-related sclerosis or granulation tissue that enhances after contrast injection. Although there are still no standardized ADC thresholds, Soldatos et al. investigated 23 patients with malignant STT subjected to neoadjuvant therapy, reporting a minimum ADC > 2.0 × 10^−3^ mm^2^/s (100% sensitivity, 61.1% specificity) and a mean ADC > 2.2 × 10^−3^ mm^2^/s (50% sensitivity, 77.8% specificity) as optimal cut-offs for determining good response [110].

Another challenging scenario in the STT imaging is post-surgical evaluation due to contrast enhancement of both scar tissue and residual/recurrent tumor. Del Grande et al. demonstrated the improved specificity of MRI by adding low ADC to conventional imaging features of relapse (52% to 97%) [111]. These results were confirmed by Kim et al., who reported a minimum ADC of 1.16 × 10^−3^ mm^2^/s, a mean ADC of 1.38 × 10^−3^ mm^2^/s, and a maximum ADC of 1.61 × 10^−3^ mm^2^/s in residual STT after unplanned resection [112]. Nevertheless, authors also highlighted the lower accuracies of DWI metrics (61.7% minimum ADC, 72.8% mean ADC, 75.6% maximum ADC) compared to those of DCE-MRI parameters (89% K_trans_, 95.1% K_ep_, 90.9% iAUC) [112].

In Table 1 we have resumed the main papers discussed in the review and their findings.

## 15. Conclusions and Future Perspectives

IVIM is emerging as an advanced DWI technique to differentiate molecular diffusion and blood microcirculation (perfusion) that lead to DWI signal. Indeed, low b values images, up to 200 s/mm^2^, are sensitive to perfusion rather than diffusion and might provide helpful information about perfusion-related features of the tumor, such as vessel density, with a non-negligible impact on the characterization, prognosis, and therapeutic response monitoring of several tumors. Further, DWI histogram analysis is certainly an interesting perspective in oncology already tested in several conditions to depict the number and distribution of pixels having the same intensity in the whole image. Basically, the heterogeneity of the histogram should reflect tumor heterogeneity, which is associated with aggressive behavior and poor response to therapies. Over the last few years, novel DKI applications outside the brain have been investigated. By acquiring DWI at ultrahigh *b*-values and post-processing images with dedicated software that provides K_app_ and D_app_ maps, DKI might help to identify the heterogeneity and irregularity of cellular microstructures with more advanced mathematical curve fitting, allowing us to explore new frontiers of imaging in the effort to overcome the existing limits of conventional DWI measurements. DWI radiomics for texture and shape feature evaluation, combined with machine learning methods, have been increasingly applied to investigate DWI’s diagnostic and prognostic roles in several tumors; this evaluation is based on the automated extraction of a number of image features beyond human perception, which can be “seen” only by computers. This line of research will probably represent the future direction for providing an objective evaluation of medical images. Indeed, the striking potential of this sequence will still be subjected to investigation to improve the correlation with underlying tumor pathology and biology, to provide a deeper understanding of molecular profile of tumors through imaging, and to allow for the construction of improved prognostic or predictive models.

## Figures and Tables

**Table 1:** Synoptic table summarizing the main papers discussed in the review and their findings.

Field	Study	Methodology	Findings
**Neuroradiology**	Yamasaki, F.; et al. Radiology. 2005 [3]	Retrospective; 275 patients with brain tumors; 1.5T MRI.	Mean ADC values are useful for differentiating brain tumors such DNT (2.546 × 10^−3^ mm^2^/s) and malignant lymphomas (0.725 × 10^−3^ mm^2^/s) versus glioblastomas (1.079 × 10^−3^ mm^2^/s) and metastatic tumors (1.149 × 10^−3^ mm^2^/s), and ependymomas (1.230 × 10^−3^ mm^2^/s) versus PNETs (1.079 × 10^−3^ mm^2^/s).
**Head and Neck**	Srinivasan, A.; et al. AJNR. Am. J. Neuroradiol, 2008 [8]	Retrospective; 33 patients (17 benign and 16 malignant lesions); 3.0T MRI.	Significant difference (*p* = 0.004) between mean ADC (10^−3^ mm^2^/s) of benign (1.505 ± 0.487) and malignant lesions (1.071 ± 0.293). Suggested threshold for differentiating benign vs. malignant = 1.3 × 10^−3^ mm^2^/s.
**Chest**	Cakir, C.; et al., Balkan. Med. J, 2015 [18]	Prospective; 48 solitary pulmonary nodules/masses (18 benign, 30 malignant); 1.5T MRI.	Mean ADC of malignant lesions (1.195 × 10^−3^ mm^2^/s) was significantly lower than that of benign lesions (2.02 × 10^−3^ mm^2^/s). ADC cut-off value of 1.5 × 10^−3^ mm^2^/s with sensitivity = 86.7% and specificity = 88.9% to differentiate benign from malignant lesions.
**Breast**	Choi, S.Y.; et al. Br. J. Radiol. 2012 [25]	Retrospective; 335 patients with IDC NOS and DCIS; 1.5T MRI.	Mean ADC of IDC NOS (0.907 ± 0.160 × 10^−3^ mm^2^/s) was significantly lower than that of DCIS (1.113 ± 0.231 × 10^−3^ mm^2^/s). Mean ADC of ER-positive cancers was significantly lower than in ER-negative.
**Hepatobiliary**	Miller, F.H.; et al. J. Magn. Reson. Imaging 2010 [32]	Retrospective; 542 focal liver lesions in 382 patients; 1.5T MRI.	Mean ADC (10^−3^ mm^2^/s) of hemangiomas = 2.26, FNH = 1.79, adenomas = 1.49, abscesses = 1.97, HCC = 1.53, and metastases = 1.50. Mean ADC of benign lesions = 2.50, malignant lesions = 1.52. Overlap reported between solid benign and malignant lesions.
**Pancreas**	Kartalis, N.; et al. Eur. Radiol. 2009 [42]	Retrospective; 36 patients with pancreatic lesions (12 malignant, 24 benign); 1.5T MRI.	DWI sensitivity = 92%, specificity = 97%, accuracy = 96%, PPV = 85%, NPV = 98%. Mean ADC (10^−3^ mm^2^/s) of malignant lesions (1.40 ± 0.30) was significantly lower than that of benign (2.57 ± 1.17).
**Esophago-Gastro-Intestinal**	Giganti, F.; et al. Radiology, 2015 [47]	Prospective study; 99 biopsy-proved gastric cancers; 1.5T MRI	ADC ≤ 1.5 × 10^−3^ mm^2^/s was associated with negative prognosis. Low ADC is a strong independent prognostic factor of the aggressiveness of gastric cancer.
**Gynecological**	Thomassin-Naggara, I. et al. Radiology. 2011 [60]	Retrospective; 87 women with complex adnexal masses (excluding endometriomas and cystic teratomas); 1.5T MRI.	High signal intensity within the solid component at *b* = 1000 s/mm was among the significant features predictive of malignancy. The use of DWI sequences (combined to perfusion) improved the accuracy to characterize complex adnexal masses
**Urinary System and Adrenal**	Ding, Y.; et al. Eur. Radiol. 2019 [67]	Retrospective; 180 patients with renal tumors (ccRCC, non-ccRCC, benign tumors); 1.5T MRI.	Mean ADC (10^−3^ mm^2^/s) of ccRCCs (1.78 ± 0.29) was significantly higher than that of non-RCC (1.31 ± 0.34) and benign renal tumors (1.35 ± 0.29). Non-ccRCCs and benign tumors had not significantly different ADC (*p* > 0.05).
**Prostate**	Woo, S.; et al. Am. J. Roentgenol. 2018 [84]	Meta-analysis including 20 studies (2242 patients with histologic-proven Prostate cancer); 1.5T and 3.0T MRI.	Biparametric MRI (T2-w and DWI) and multiparametric MRI (T2-w, DWI, and DCE) had not significantly different diagnostic performance (pooled sensitivity and specificity of 0.74 and 0.90 vs. 0.76 and 0.89, respectively).
**Lymph Nodes and Spleen**	Albano, D.; et al., Eur. J. Radiol. 2016 [87]	Prospective; 68 patients with FDG-avid lymphoma. Comparison between WB-MRI with DWI and FDG-PET/CT. 1.5T MRI.	Excellent agreement between WB-MRI and FDG-PET/CT stage (k = 0.88; *p* < 0.05); WB-MRI stage was equal to FDG-PET/CT stage in 91.2% (62/68; in particular, 35/37 Hodgkin lymphoma, 27/31 Non Hodgkin lymphoma). Sensitivity of WB-MRI for splenic involvement was 100%.
**Bone Tumors**	Wang, T.; et al. World J. Surg. Oncol. 2014 [96]	Retrospective; 187 patients; 3.0T MRI.	Mean ADC (×10^−3^ mm^2^/s) of benign tumors (1.17 ± 0.36) significantly higher than that of malignant (0.87 ± 0.20).
**Soft Tissue Tumors**	Choi, Y.J.; et al. J. Magn. Reson. Imaging 2019 [107]	Retrospective; 136 patients; 3.0T MRI.	Significant difference between mean ADC (×10^−3^ mm^2^/s) of benign (1.44 ± 0.46) and malignant (0.9 ± 0.40) soft tissue tumors.

Note—DNT = dysembryoplastic neuroepithelial tumors; IDC NOS = invasive ductal carcinoma not otherwise specified; DCIS = ductal carcinoma in situ; ER = estrogen receptor; FNH = focal nodular hyperplasia; HCC = hepatocellular carcinoma; PPV = positive predictive values; NPV = negative predictive values; ccRCC = clear cell renal cell carcinoma; FDG = fluorodeoxyglucose; WB-MRI = whole body MRI.

## Abbreviations

DWI	Diffusion weighted imaging
MRI	Magnetic Resonance Imaging
ADC	Apparent diffusion coefficient
HGGs	High-Grade Gliomas
IVIM	Intravoxel incoherent motion
DKI	Diffusional kurtosis imaging
HNT	Head and neck tumor
HNSCC	Head and neck squamous cell carcinoma
STIR	Short tau inversion recovery
DCE	Dynamic contrast enhanced
HCC	Hepatocellular carcinoma
PPV	Positive predictive value
NPV	Negative predictive value
pNENs	Pancreatic neuroendocrine neoplasms
ccRCC	Clear cell renal cell carcinoma
mpMRI	Multi-parametric MRI
PCa	Prostate cancer
PI-RADS	Prostate imaging reporting and data system
WB	Whole-body
DWIBS	DWI with background suppression
BT	Bone tumors
STT	Soft tissue tumors
